# Unraveling metabolic reprogramming in *Δhnox* *Paracoccus denitrificans*: a time-resolved metabolomics and AI-Powered proteome modeling approach

**DOI:** 10.3389/fmolb.2025.1679650

**Published:** 2025-10-21

**Authors:** Md Shariful Islam, Aishat Alatishe, William Bahureksa, Erik Yukl

**Affiliations:** ^1^ Department of Chemistry and Biochemistry, New Mexico State University, Las Cruces, NM, United States; ^2^ Chemical Analysis and Instrumentation Laboratory Research Cores Program, New Mexico State University, Las Cruces, NM, United States

**Keywords:** metabolomics, proteomics, biofilm, artificial intelligence - AI, H-NOX

## Abstract

Heme-nitric oxide/oxygen binding (H-NOX) proteins function as critical sensors for nitric oxide in many bacterial species. However, their physiological functions are surprisingly diverse, and most have yet to be thoroughly investigated. Here, we investigate the impact of *hnox* deletion in *Paracoccus denitrificans*, a species known for its metabolic versatility and the formation of unusually thin biofilm structures. Time-resolved targeted metabolomics across three growth phases (OD_600_ = 0.6, 2.0, and 4.0) indicates that the deletion of *hnox* is consistently associated with disruptions in central carbon metabolism. At early stages, the *Δhnox* strain exhibits increased abundance of glycolytic and pentose phosphate pathway metabolites accompanied by decreases in amino acids, suggesting dysregulation in late glycolysis or promotion of fermentative metabolism. Higher cell densities are characterized by increased quorum sensing, which is shown to promote biofilm dispersal in the WT but had little effect on the *Δhnox* strain. Metabolomics changes at these stages continue to highlight the pentose phosphate and glycolytic metabolites along with redox cofactors, implicating changes in energy metabolism or oxidative stress response. Total proteomics at OD_600_ = 2.0 were collected to explore connections between metabolism and proteome dynamics, and to provide an opportunity to test several machine learning (ML) models for predicting proteomic changes from metabolomic profiles. While constrained by limited sample size, these exploratory models showed biologically meaningful concordance with experimentally observed proteome shifts, highlighting both the promise and the current limitations of artificial intelligence (AI)-based methods in non-model microbial systems.

## Introduction

*Paracoccus* *denitrificans* is a Gram-negative, facultatively anaerobic bacterium capable of complete denitrification, a biochemical process that entails the sequential reduction of nitrate to nitrogen gas via intermediates such as nitrite, nitric oxide, and nitrous oxide (Baker et al., 1998; Xia et al., 2018; Stouthamer et al., 1997). Understanding this process is crucial for addressing the buildup of harmful nitrogenous compounds in the environment and has important implications for wastewater management and environmental engineering (Pascual et al., 2020; Assefa et al., 2019; Strauss et al., 2012). Furthermore, *P. denitrificans* possesses the potential for a range of biotechnological applications, including bioremediation and the enhancement of sustainable agricultural practices (Puri et al., 2022; Yang et al., 2013; Kojima et al., 2004; Lycus et al., 2018; Gómez-Acata et al., 2018; Zhao et al., 2015; Olaya-Abril et al., 2018; Islam et al., 2024).

Practical application of *P. denitrificans* in these processes may be improved by or even require the formation of a monoculture or mixed-species biofilms (Nisha et al., 2015; Singh et al., 2015; Kiely et al., 2010). Biofilms are structured communities of cells encased within a self-produced extracellular polymeric substance (EPS), which confers tolerance to antimicrobials and various environmental stresses such as changes in pH, mechanical shear, osmolarity, and nutrient availability (Mishra et al., 2023). *P. denitrificans* is noted for its unusually thin biofilms consisting of densely packed cells in what is nearly a monolayer (Yoshida et al., 2017; Singh et al., 2015), which contrasts with the large “mushroom” structures of EPS and cells formed by well-studied biofilm producers such as *Pseudomonas aeruginosa* (Sauer et al., 2002; Sauer et al., 2022). The processes of surface adhesion and EPS production during the planktonic to sessile transition are tightly regulated by environmental cues and both inter- and intracellular signaling pathways, although these may differ substantially between species (Costerton et al., 1999; Flemming and Wingender, 2010; Flemming et al., 2016). In *P. denitrificans*, biofilm formation requires the calcium-dependent adhesin BapA (Kumar and Spiro, 2017; Yoshida et al., 2017) and is regulated through a quorum sensing (QS) circuit with enzymes PdeI and PdeR acting as acylhomoserine lactone (AHL) synthase and transcription factor/response regulator, respectively (Zhang et al., 2018). Deletion of *pdeI* promoted biofilm formation while exogenous addition of C16 AHL inhibited it, suggesting an inhibitory role for QS in this organism (Morinaga et al., 2018). However, *pdeR* overexpression was shown to promote biofilm formation (Wang N. et al., 2022), perhaps suggesting distinct roles for PdeR alone and in complex with AHL. There is also data suggesting that cyclic diguanosine monophosphate (c-di-GMP) inhibits biofilm formation as deletion of the two annotated diguanylate cyclase (DGC) genes results in increased biofilm formation (Kumar and Spiro, 2017). This is unusual in that c-di-GMP generally signals for biofilm development (Römling et al., 2013).

Adjacent to one of the DGC genes (*dgcA*) is a gene encoding a heme-nitric oxide/oxygen binding protein (H-NOX). H-NOX proteins are known to modulate communal behaviors such as biofilm formation and quorum sensing in response to NO, often acting upstream of cyclic-di-GMP regulatory pathways (Plate and Marletta, 2013). Indeed, the *Δhnox* mutant of *P. denitrificans* displays a markedly biofilm-deficient phenotype and elevated levels of AHL relative to the WT at high cell densities (Kumar and Spiro, 2017; Islam et al., 2024). Moreover, a comprehensive proteomics study of *Δhnox P. denitrificans* showed altered abundance of proteins involved in central carbon and energy metabolism occurring at cell densities (OD_600_ = 0.6) that precede dysregulation of AHL (Islam et al., 2024). These findings suggest a role for H-NOX in coordinating metabolism with biofilm formation in this organism.

In the present study, we explored this regulatory role more deeply by focusing on how core metabolic pathways—including glycolysis, the pentose phosphate pathway (PPP), and the tricarboxylic acid (TCA) cycle—contribute to biofilm development in *P. denitrificans.* We performed targeted metabolomic profiling comparing WT and *Δhnox P. denitrificans* across different stages of bacterial growth, which revealed changes in the abundance of metabolites associated with amino acid metabolism, glycolysis, and PPP. Furthermore, by integrating metabolomic and proteomic data from cultures at OD_600_ = 0.6, we employed an AI learning framework to successfully predict protein expression profiles at OD_600_ = 2. The application of supervised ML algorithms and graph neural networks (GNN) predicted correlations between metabolite abundance and protein output that were largely verified by mass spectrometry. AI-based methodologies have demonstrated growing effectiveness in the field of systems biology, especially in the modeling of high-dimensional omics data, the inference of regulatory interactions, and the prediction of phenotypic outcomes in response to genetic or environmental perturbations (Camacho et al., 2018; Ma et al., 2018; Ching et al., 2018). Collectively, our data suggest that *hnox* deletion induces alterations in metabolic regulation, disrupting biofilm development at low cell densities and manifesting persistent changes in energy metabolism and oxidative stress response at high cell densities.

## Materials and methods

### Bacterial strains and culture conditions

WT and *Δhnox P. denitrificans* PD1222 strains were a kind gift from Dr. Stephen Spiro at the University of Texas Dallas (Kumar and Spiro, 2017). As noted below, strains were grown in LB medium (Formula/liter: Tryptone 10 g, Yeast extract 5 g and Sodium Chloride 10 g) with or without supplementation with 10 mM CaCl_2_ at 30 °C with shaking at 50–100 rpm.

### Bacterial growth and crystal violet staining

For growth experiments, overnight cultures in LB medium were diluted to OD_600_ = 0.05 in 12 mL of fresh LB in 50 mL plastic culture tubes without CaCl_2_ supplementation to match conditions used for metabolomics samples (see below). Three replicate samples were maintained at 30 °C with shaking at 100 rpm and OD_600_ was measured every 4 h. Crystal violet (CV) staining experiments were performed in the same way except that LB was supplemented with 10 mM CaCl_2_ and shaking was at 50 rpm to facilitate biofilm formation (Kumar and Spiro, 2017). Samples were monitored until they reached the desired OD_600_ (3 replicates for each OD), at which point media and planktonic cells were discarded, and the tubes were rinsed 3 times with deionized water and air dried. 12 mL of 0.1% w/v CV was added and shaken at room temperature for 1 h. Stain was discarded, and the tubes were again rinsed 3 times with deionized water and allowed to dry. Finally, CV was dissolved with 12 mL of 100% DMSO and absorbance at 570 nm was measured.

### Scanning electron microscopy (SEM) sample preparation and imaging

Overnight starter cultures were adjusted to OD_600_ = 0.05 in 3 mL of fresh LB medium supplemented with 10 mM CaCl_2_ in sterile polystyrene Petri plates, each bearing a pre-cleaned glass coverslip. Cultures were maintained at 30 °C with shaking at 50 rpm for 24 h to facilitate surface adhesion and biofilm development. Following incubation, planktonic cultures were discarded, and the coverslips were rinsed three times with deionized water to eliminate non-adherent cells. Coverslips were subsequently air-dried under sterile conditions for 48 h. Thereafter, samples were subjected to a 10-min treatment with a drop of 2% ionic liquid, 1-butyl-3-methylimidazolium tetrafluoroborate (Sigma, St. Louis, Mo, United States), to improve conductivity, and surplus liquid was blotted with Kimwipes. The dried and processed samples were sputter-coated with gold utilizing a Gatan Model SSI High Resolution Ion Beam Coater (Gatan, Pleasanton, California, United States). Imaging was conducted utilizing a Hitachi SU7000 Field Emission Scanning Electron Microscope (Hitachi, Japan). All assays were performed in triplicate for both WT and *Δhnox* strains.

### Sample preparation for targeted metabolomics analysis

For metabolomics experiments, CaCl_2_ supplementation was omitted as excess Ca^2+^ may interfere with ion-pair chromatography and electrospray ionization in the triple quadrupole LC-MS/MS platform. WT and *Δhnox P. denitrificans* starter cultures were adjusted to OD_600_ = 0.05 in LB media and maintained at 30 °C with shaking at 100 rpm. Cells were harvested at OD_600_ = 0.6, 2.0, or 4.0, which required approximately 18–72 h growth under these conditions (Supplementary Figure S1). Four biological replicate samples were used at each cell density. Cells were collected using centrifugation at 4,300 × g for 20 min at 4 °*C. Media* was carefully removed by pipette, and the cell pellets were immediately weighed and subjected to three washes with 1 mL of ice-cold phosphate-buffered saline (PBS) immediately followed by resuspension in ice-cold methanol containing internal standard (10 μmol L^-1^ of L-methionine sulfone). Samples were homogenized by sonication for 2 min, followed by three freeze-thaw cycles in liquid nitrogen to guarantee complete extraction. Samples were centrifuged at 12,000 × g for 10 min at 4 °C and supernatants were transferred to 15 mL conical tubes. Phase separation was executed by incorporating 0.5 mL of deionized water and 1 mL of chloroform into each sample followed by centrifugation at 4,000 × g for 15 min at 4 °C. The top aqueous phase was extracted for subsequent processing. The samples were filtered using pre-washed 3-kDa molecular weight cutoff filters (Millipore, Tokyo, Japan) via centrifugation to eliminate protein impurities. The filtrates were lyophilized overnight at 4 °C and subsequently kept at −80 °C until analysis. Dried metabolites were reconstituted in 100 μL of deionized water for triple quadrupole mass spectrometry analysis. Metabolite concentrations were adjusted according to cell pellet wet weight.

### Triple-quadrupole mass spectrometry analysis

The analysis method used was the Primary Metabolites LC/MS/MS method package developed by Shimadzu with some modifications (Kubo et al., 2011). The Primary Metabolites package utilizes an ion-pairing reagent (i.e., tributylamine) in order to enhance retention of metabolites and an internal standard (L-methionine sulfone) to perform semi-quantitative analysis between the WT and *Δhnox* samples. Extracts were analyzed using a Shimadzu LCMS-8050 instrument which couples liquid chromatography (LC) with a triple-quadrupole mass spectrometry (MS) instrument (Shimadzu Scientific Instruments, Columbia, MD, United States). The LC column was the Mastro 2 (Shimadzu, C18, 3 μm, 150 × 2 mm) and mobile phases were water (A: 15 mM acetic acid, 5 mM tributylamine-water) and methanol (B). The LC gradient program was as follows: 0% B (0.5 min) - 25% B (8.0 min) - 98% B (12.0–15.0 min) - 0% B (15.1–20.0 min). Flow rate was 0.30 mL/min and the column temperature was kept at 40 °C during each run. Nebulizing and drying gas were kept at 2.0 and 10 L/min, respectively. The desolvation line and heat block temperatures were kept at 250 °C and 400 °C, respectively. The LCMS-8050 was operated using negative (−) mode electrospray ionization with multiple-reaction-monitoring (MRM) for each of the compounds as described in the Primary Metabolites package (Kubo et al., 2011).

### AI-based proteome prediction

To predict protein expression at OD_600_ = 2 for both WT and *Δhnox P. denitrificans*, we used a data set of metabolomics profile at OD_600_ = 0.6 and 2.0, as well as proteomics data at OD_600_ = 0.6 (Islam et al., 2024). Supervised ML models were trained on this multi-omics dataset to quantify metabolite abundance and proteome expression. ML models like Partial Least Squares (PLS), Ridge regression, Lasso regression, Random Forest, and XGBoost were used in the current study (Samin et al., 2024; Greener et al., 2022). For comparability, all features were scaled before model training. Leave-one-out cross-validation (LOOCV) was used to evaluate each model’s prediction performance under limited-sample settings. For the classical machine learning models (Partial Least Squares, Ridge, Lasso, Random Forest, XGBoost), we used the scikit-learn (v1.2.2) and XGBoost (v1.7.6) implementations with the following specifications: Data preprocessing: all features were scaled (standardization for proteomics, MinMax scaling for metabolomics). Cross-validation: Leave-One-Out Cross Validation (LOOCV). Ridge/Lasso: optimized using default coordinate descent solvers; regularization parameters were tuned using grid search. Random Forest: 100 estimators, Gini criterion, max depth unrestricted. XGBoost: learning rate 0.1, max depth 6, 200 estimators. For the graph neural network (GNN) framework (PyTorch Geometric v2.3.1), we implemented a two-layer Graph Attention Network (GAT) with hidden dimension of 128 and four attention heads per layer. Batch Normalization and dropout layers (dropout rate = 0.3) were included to improve generalization. The Exponential Linear Unit (ELU) activation function was used throughout. Models were trained with the Adam optimizer (learning rate = 0.001), coupled with a StepLR scheduler (step size = 100, γ = 0.5). Mean Squared Error (MSE) was employed as the loss function, and training proceeded for 300 epochs until convergence, as monitored by loss stabilization with no signs of overfitting. The GNN architecture explicitly incorporated topological linkages among biological entities, thereby capturing context-dependent interactions and pathway-level organization within the metabolomics-proteomics network (Scarselli et al., 2009). All ML analyses were performed in Python 3.10.12 on Google Colab, a cloud-based environment for reproducible and scalable computation (Bisong, 2019). The following package versions were used: NumPy 1.23.5; pandas 1.5.3; scikit-learn 1.2.2; PyTorch 2.0.1; PyTorch Geometric 2.3.1; torch-scatter 2.1.1; torch-sparse 0.6.17; XGBoost 1.7.6; Matplotlib 3.7.1; and Seaborn 0.12.2. *Scikit-learn* and *XGBoost* supported data preprocessing and classical ML training, while *PyTorch* and *PyTorch Geometric* were used for GNN implementation. Model performance and predictions were visualized with Matplotlib and Seaborn. Links to the code used are provided in the Data Availability section.

### Proteomics sample preparation and data analysis

WT and *Δhnox P. denitrificans* PD1222 were grown in 10 mL LB media supplemented with 10 mM CaCl_2_ at 30 °C with shaking at 100 rpm until OD_600_ reached 2.0. At this point, cells were collected via centrifugation, rinsed, lysed by sonication in buffer containing 6 M urea, 2 M thiourea, 25 mM NH_4_HCO_3,_ and protease inhibitor, and subsequently cleared using centrifugation to isolate soluble proteins. Protein extraction and tryptic digestion were conducted according to a previously established methodology (Cox et al., 2014). Proteomic analyses were performed on four independent biological replicates per condition, without additional technical replication. The resultant peptides were examined utilizing an Orbitrap Eclipse Tribrid mass spectrometer (Thermo Fisher Scientific, Waltham, MA, United States). RAW proteome data were analyzed with MaxQuant (v2.2.0.0) against the *P. denitrificans* UniprotKB database (UP000000361) (Cox and Mann, 2008). Carbamidomethylation of cysteine was designated as a permanent modification, whereas oxidation of methionine and N-terminal acetylation were classified as variable modifications. A 1% false discovery rate threshold was implemented for the identification of peptides and proteins. Data visualizations were executed in Python.

### Statistical analysis

All metabolomics and proteomics data were subjected to analysis utilizing Python 3.10, along with the libraries NumPy, pandas, Matplotlib, and Seaborn. Metabolite and protein comparisons between WT and *Δhnox* strains were conducted using two-tailed Student’s t-tests. Metabolites were considered significant if they exhibited p < 0.05 (Graf et al., 2019; Wang R. et al., 2022; Wang et al., 2025) and an absolute log_2_ fold change ≥0.58, which corresponds to a 1.5-fold change. In addition, Storey’s *q*-values (false discovery rate estimates) were computed and are presented alongside the raw *p*-values in the result tables for reference but were not used as the primary basis for determining statistical significance. The metabolomics and proteomics datasets were each generated from four independent biological replicates per condition. For proteomics, a 1% false discovery rate (FDR) threshold was applied for peptide and protein identification in MaxQuant (v2.2.0.0). For machine learning analyses, all features were scaled, leave-one-out cross-validation (LOOCV) was used, and model performance was assessed by mean squared error (MSE). Figures were generated using Python libraries Matplotlib and Seaborn. For pathway-level interpretation, significantly altered metabolites (normalized and scaled) were projected onto PCA-based biplots using pathway annotations to visualize clustering and separation of metabolites by functional categories.

## Results

### *P. denitrificans* *Δhnox* is biofilm deficient

**FIGURE 1:**
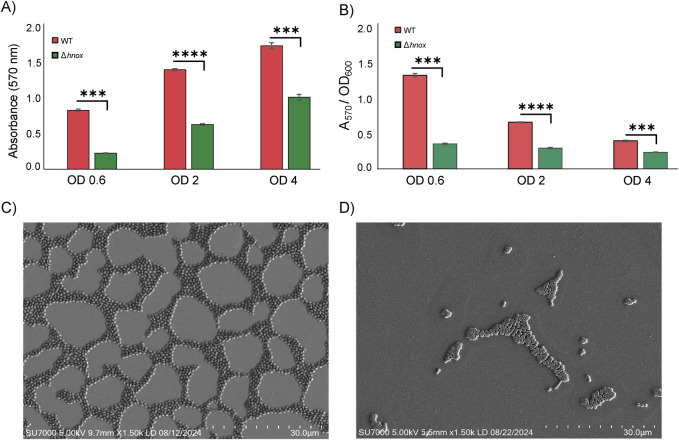
Biofilm formation in WT and *Δhnox P. denitrificans*. CV staining for cultures at different cell densities **(A)** as raw data and **(B)** normalized by OD_600_. Error bars represent standard deviation (n = 3; ***p < 0.01 and ****p < 0.0001 by Student’s *t* test for pairwise comparisons). SEM images for surface-attached **(C)** WT and **(D)** *Δhnox P. denitrificans*. Imaging was performed using the SU7000 Field Emission SEM and the scale bar represents 30 µm.

Previous work showed that deletion of *hnox* had no significant effect on *P. denitrificans* growth in microplates but essentially abrogated its ability to form biofilms in 50 mL plastic culture tubes at OD_600_ = 0.6 (Islam et al., 2024). Here we have expanded to monitor growth and biofilm formation across cell densities ranging from OD_600_ = 0.6–4.0. The *hnox* deletion had no influence on growth in 50 mL plastic culture tubes (Supplementary Figure S1), but it significantly impaired biofilm formation at all cell densities tested (Figure 1A). However, normalizing CV staining by OD_600_ reveals that relative biofilm formation in the WT is greatest at OD_600_ = 0.6 and decreases with increasing cell density. This is roughly anticorrelated with C16 AHL concentration (Islam et al., 2024), consistent with previous data (Morinaga et al., 2018) showing this autoinducer to be a negative regulator of biofilm in *P denitrificans*. Conversely, normalized biofilm in the *Δhnox* remains nearly constant across different cell densities (Figure 1B).

To elucidate the structural consequences of the *hnox* deletion on bacterial communities, we utilized scanning electron microscopy (SEM) to analyze and compare WT and *Δhnox P. denitrificans*. In the WT strain, we observe a highly organized network of bacterial cells with large empty regions corresponding to gaps between tightly packed microcolonies in the thin biofilm layer of *P. denitrificans* (Figure 1C). The discontinuous surface population is consistent with what has been measured by confocal microscopy (Morinaga et al., 2020), although perhaps sparser than some analyses (Yoshida et al., 2017), which may be a consequence of differences in sample preparation. Nevertheless, both techniques yield data consistent with a monolayer or near-monolayer biofilm of densely packed cells with very little evidence of an extensive EPS matrix (Wang N. et al., 2022). Conversely, very few *Δhnox* cells were found adhered to the surface and these in small, isolated microcolonies (Figure 1D). This is consistent with the markedly diminished biofilm biomass in this strain compared to WT as quantified through crystal violet staining (Kumar and Spiro, 2017; Islam et al., 2024).

### Targeted metabolomics at OD_600_ = 0.6

**FIGURE 2:**
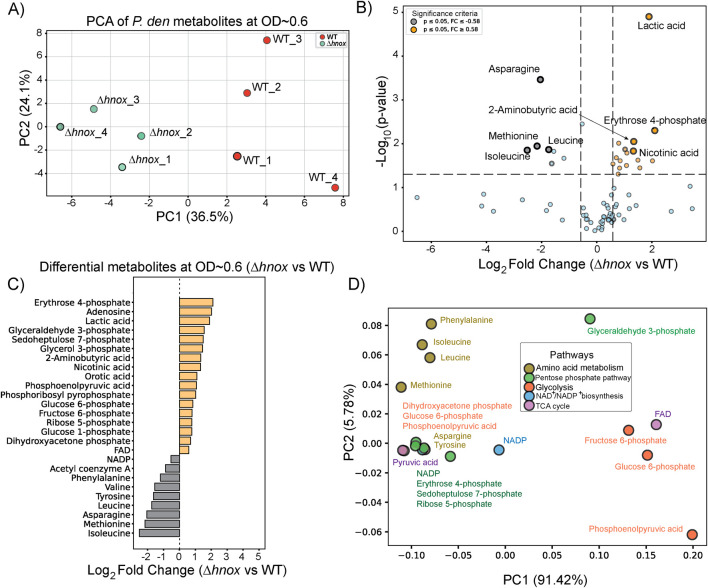
Metabolomic changes in *Δhnox P. denitrificans* at OD_600_ = 0.6. **(A)** PCA of metabolite profiles distinguishes WT and *Δhnox* samples, indicating distinct metabolic states. **(B)** Volcano plot showing the differential abundance of metabolites between *Δhnox* and WT strains. The x-axis represents the log_2_ fold change (*Δhnox*/WT), and the y-axis shows the –log_10_ (p-value). Metabolites with increased abundance in *Δhnox* vs. WT are indicated as yellow dots while metabolites with decreased abundance are indicated as gray dots. **(C)** Bar plot of relative abundance for significantly altered metabolites in *Δhnox* relative to WT colored as in B. **(D)** PCA biplot of significantly altered metabolites based on pathway annotations. Metabolites are colored by their associated pathway.

To investigate the effect of *hnox* deletion on central carbon metabolism, we performed targeted metabolomics analysis at OD_600_ = 0.6 using an ion-pairing liquid chromatography–mass spectrometry (LC-MS) method optimized for central carbon metabolites (Kubo et al., 2011). This time point was selected based on our previous proteomics study, which revealed significant changes in protein expression linked to metabolic pathways in *Δhnox P. denitrificans* (Islam et al., 2024). A total of 73 metabolites associated with central carbon metabolism were identified by this method (Supplementary Table S1). Principal Component Analysis (PCA) demonstrated clear separation between WT and *Δhnox* samples, signifying a distinct metabolic profile in the absence of *hnox* (Figure 2A). We conducted a statistical comparison between *Δhnox* and WT strains to identify metabolites with significant differential abundance as shown in the volcano plot (Figure 2B). A total of 26 metabolites showed significant changes (|log_2_ fold change| ≥ 0.58, p < 0.05). Several intermediates of the PPP and glycolysis including erythrose 4-phosphate, glyceraldehyde 3-phosphate, sedoheptulose 7-phosphate, phosphoenolpyruvic acid, glucose 6-phosphate, and fructose 6-phosphate were significantly more abundant in *Δhnox* samples (Figure 2C). In contrast, 7 amino acids (valine, leucine, tyrosine, phenylalanine, isoleucine, methionine, and asparagine) were decreased in abundance and one (aspartate) was observed only in WT samples. Acetyl coenzyme A and NADP were also decreased in abundance. Finally, pyruvic acid was consistently detected in WT samples but was undetectable in *Δhnox* samples while lactic acid abundance was significantly increased in *Δhnox* samples.

Next, we performed a metabolite PCA-based pathway projection to investigate the pathway-level organization of the altered metabolites. The significantly changed candidates were categorized into key metabolic routes, including amino acid metabolism, glycolysis, the PPP, the TCA cycle, and NAD/NADP^+^ biosynthesis (Figure 2D). The first principal component (PC1) accounts for 91.42% of the variance, effectively distinguishing metabolites associated with amino acid metabolism (e.g., asparagine, valine) and the PPP (e.g., sedoheptulose 7-phosphate, erythrose 4-phosphate) from those related to glycolysis (e.g., fructose 6-phosphate, glucose 6-phosphate) and cofactor biosynthesis (e.g., FAD, NADP^+^). PC2 accounts for 5.78% of the variance and facilitates the differentiation between amino acids and PPP metabolites. The amino acids in particular cluster distinctly from other metabolite classes, reaffirming altered amino acid metabolism in the Δ*hnox* strain.

### Targeted metabolomics at OD_600_ = 2.0

**FIGURE 3:**
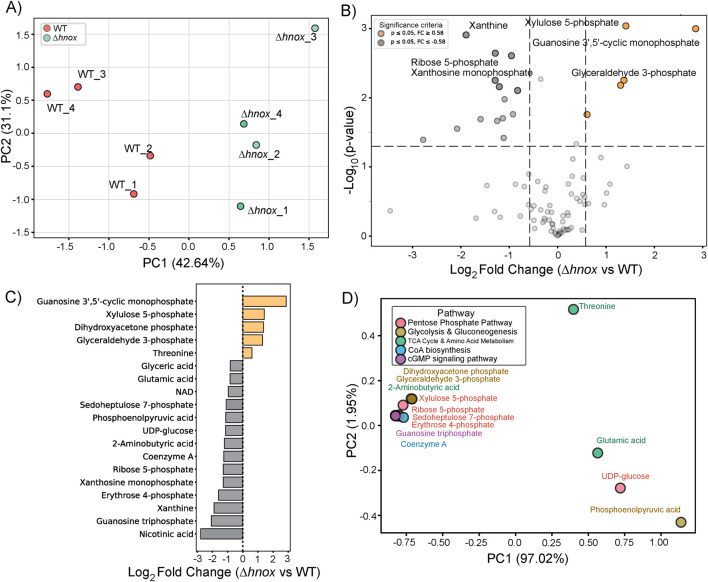
Metabolomic changes in *Δhnox P. denitrificans* at OD_600_ = 2.0. **(A)** PCA of metabolite profiles distinguishes WT and *Δhnox* samples, indicating distinct metabolic states. **(B)** Volcano plot showing the differential abundance of metabolites between *Δhnox* and WT strains. The x-axis represents the log_2_ fold change (*Δhnox*/WT), and the y-axis shows the –log_10_ (p-value). Metabolites with increased abundance in *Δhnox* vs. WT are indicated as yellow dots while metabolites with decreased abundance are indicated as gray dots. **(C)** Bar plot of relative abundance for significantly altered metabolites in *Δhnox* relative to WT colored as in B. **(D)** PCA biplot of significantly altered metabolites based on pathway annotations. Metabolites are colored by their associated pathway.

Next we conducted targeted metabolomics profiling of *P. denitrificans* at OD_600_ = 2.0. In total, 88 metabolites were detected (Supplementary Table S2). PCA revealed a distinct separation between WT and *Δhnox* samples (Figure 3A), suggesting that the deletion of *hnox* induces specific metabolic remodeling during this growth phase. The differential abundance analysis conducted via a volcano plot identified 21 metabolites with significant alterations (|log_2_ fold change| ≥ 0.58, p < 0.05) when comparing *Δhnox* to WT (Figure 3B). Abundance of glyceraldehyde 3-phosphate continues to be increased while several other intermediates from the PPP and glycolysis were modestly decreased, including sedoheptulose 7-phosphate, erythrose 4-phosphate, phosphoenolpyruvate. Additionally, we observed a reduction in coenzyme A, nicotinic acid, NAD^+^, xanthosine monophosphate, xanthine, and guanosine triphosphate (GTP) (Figure 3C). Conversely, a small subset of metabolites exhibited increased abundance in the *Δhnox* strain. These include guanosine 3′,5′-cyclic monophosphate (cGMP) and xylulose 5-phosphate.

The initial PC1 of the PCA-based biplot (Figure 3D) accounted for 97.02% of the variance and effectively categorized essential metabolites according to their respective pathway classes. Except for phosphoenolpyruvate, the intermediates of glycolysis and the PPP formed a cohesive cluster, distinct from UDP-glucose and the amino acids threonine and glutamate, which were modestly increased and decreased in abundance, respectively.

### Targeted metabolomics at OD_600_ = 4.0

**FIGURE 4:**
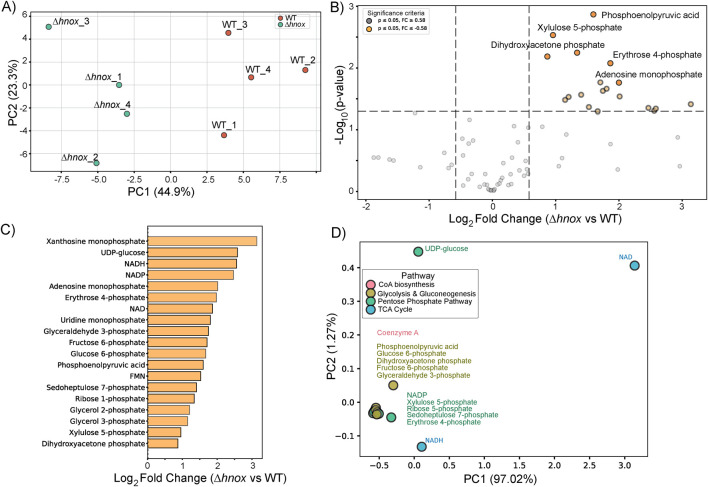
Metabolomic changes in *Δhnox P. denitrificans* at OD_600_ = 4.0. **(A)** PCA of metabolite profiles distinguishes WT and *Δhnox* samples, indicating distinct metabolic states. **(B)** Volcano plot showing the differential abundance of metabolites between *Δhnox* and WT strains. The x-axis represents the log_2_ fold change (*Δhnox*/WT), and the y-axis shows the –log_10_ (p-value). Metabolites with increased abundance in *Δhnox* vs. WT are indicated as yellow dots while metabolites with decreased abundance are indicated as gray dots. **(C)** Bar plot of relative abundance for significantly altered metabolites in *Δhnox* relative to WT colored as in B. **(D)** PCA biplot of significantly altered metabolites based on pathway annotations. Metabolites are colored by their associated pathway.

To examine the impact of *hnox* deletion on metabolism during extended growth periods, we performed targeted metabolomics profiling of *P. denitrificans* at OD_600_ = 4.0. A total of 80 metabolites were identified in both WT and *Δhnox* strains (Supplementary Table S3). PCA demonstrated a global separation between WT and *Δhnox* samples (Figure 4A). The profile at OD_600_ = 4.0 was distinctly marked by the upregulation of all significantly altered metabolites in the *Δhnox* mutant relative to the WT (Figure 4B). Intermediates of PPP and glycolysis were again identified as were the redox active cofactors NAD(H), NADPH, and FMN, as well as the nucleotide monophosphates xanthosine monophosphate (XMP) and adenosine monophosphate (AMP) (Figure 4C). The biplot (Figure 4D) demonstrated that PPP and glycolytic intermediates, such as sedoheptulose 7-phosphate, erythrose 4-phosphate, and DHAP, formed a close cluster suggesting coordinated regulation of these pathways.

### The global impact of *hnox* deletion on metabolism across different growth phases

**FIGURE 5:**
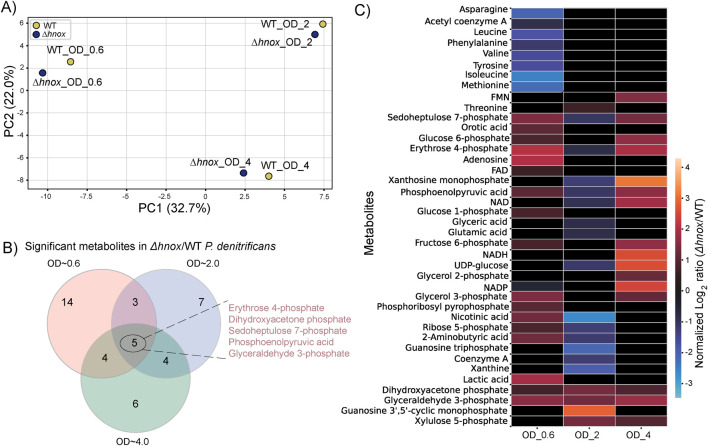
Comparative metabolomic analysis across growth phases in *Δhnox* vs. WT *P. denitrificans*. **(A)** PCA of metabolite profiles of WT and *Δhnox* at OD_600_ = 0.6, 2.0, and 4.0. **(B)** Venn diagram of significantly altered metabolites in *Δhnox* versus WT across all three ODs. **(C)** Heatmap showing normalized log_2_ fold changes of metabolites significantly altered at OD_600_ = 0.6, 2.0 or 4.0.

Figure 5 combines the metabolomic profile data from WT and *Δhnox P. denitrificans* at OD_600_ = 0.6, 2.0, and 4.0. PCA demonstrates a clear distinction between samples at varying ODs (Figure 5A), signifying divergent metabolic states as cell density increases. Although *Δhnox* and WT samples were distinguishable at every time point, their relative locations exhibited a similar pattern throughout the development phases. This indicates that global metabolic changes are driven by growth phase with more subtle differences dependent upon *hnox* status.

A Venn diagram of all significantly modified metabolites across all three growth phases (Figure 5B) shows that five metabolites—erythrose 4-phosphate, glyceraldehyde 3-phosphate, phosphoenolpyruvic acid, sedoheptulose 7-phosphate, and dihydroxyacetone phosphate—were consistently differentially abundant at all three optical densities. Other metabolites were modified only at certain growth phases. Figure 5C shows normalized log_2_ fold changes of significantly changed metabolites across cell densities to reveal phase-specific expression patterns.

### Proteomics at OD_600_ = 2.0

**FIGURE 6:**
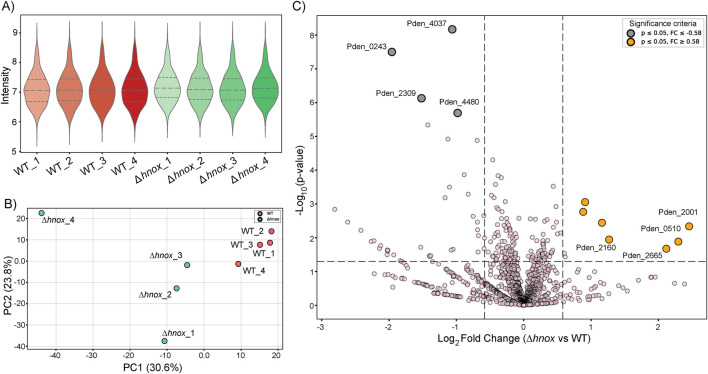
Proteomics comparison of *Δhnox* vs. WT *P. denitrificans* at OD_600_ = 2.0. **(A)** Violin plots showing the distribution of protein intensity values across biological replicates of WT (n = 4) and *Δhnox* (n = 4) strains. Median and quartile ranges are indicated by dashed lines. **(B)** PCA plot illustrating separation of WT (red) and *Δhnox* (green) proteome profiles. PC1 (30.6%) and PC2 (23.8%) capture the major variance between conditions. **(C)** Volcano plot comparing *Δhnox* versus WT proteomes. The x-axis represents log_2_ fold change, and the y-axis represents–log_10_ p-value. Significantly downregulated proteins (p ≤ 0.05, FC ≤ −0.58) are shown in gray, while significantly upregulated proteins (p ≤ 0.05, FC ≥ 0.58) are shown in orange. Selected candidate proteins are labeled.

To further enable correlation of metabolomic and proteomic changes across cell densities, we performed total proteomics at OD_600_ = 2.0 to add to our existing dataset at OD_600_ = 0.6 (Islam et al., 2024). A label-free approach identified more than 1,600 proteins with 35 differentially expressed between WT and *Δhnox* strains (Supplementary Table S4; Figure 6).

The data quality and distribution of protein intensities were first assessed using violin plots, as shown in Figure 6A. Both WT and *Δhnox* samples displayed comparable distributions with overlapping ranges, confirming consistent protein detection and reproducibility. PCA was then applied to capture global variation in proteome profiles (Figure 6B). The first two principal components accounted for more than 50% of the variance, and samples clustered tightly within their respective groups, while WT and *Δhnox* were clearly separated along PC1, indicating distinct strain-specific proteomic signatures. Finally, volcano plot analysis was performed to highlight proteins showing significant changes between the two strains (Figure 6C). Several proteins including Pden_4037, Pden_0243, Pden_2001 and Pden_0510 with strong statistical support and high fold changes were annotated directly on the plot, underscoring candidate proteins most affected by H-NOX deletion.

### Prediction of proteome expression at OD_600_ = 2.0 using multi-OMICs integration and artificial intelligence-based models

Implementing our current established dataset, which included metabolomics profiles at OD_600_ = 0.6 and 2.0, alongside proteomics data at OD_600_ = 0.6 (Islam et al., 2024), we aimed to forecast differentially expressed proteins at an OD_600_ = 2.0 in *Δhnox P. denitrificans* relative to the WT strain. We applied various supervised ML models, including PLS, Ridge regression, Lasso regression, Random Forest, and XGBoost, to establish predictive relationships between metabolite levels and protein expression. LOOCV was used to evaluate the performance of the model. Of all the models evaluated, Ridge regression yielded the lowest mean squared error, thereby establishing it as the most effective method in this context (Supplementary Figure S2). Models such as PLS and Random Forest exhibited satisfactory performance. However, XGBoost demonstrated increased variability and error, indicating that it may not be optimally suited for our dataset in its present configuration. Alongside the conventional ML models, we also employed a GNN, which is a deep learning architecture specifically developed to leverage the relationships and structural characteristics inherent in biological data.

**FIGURE 7:**
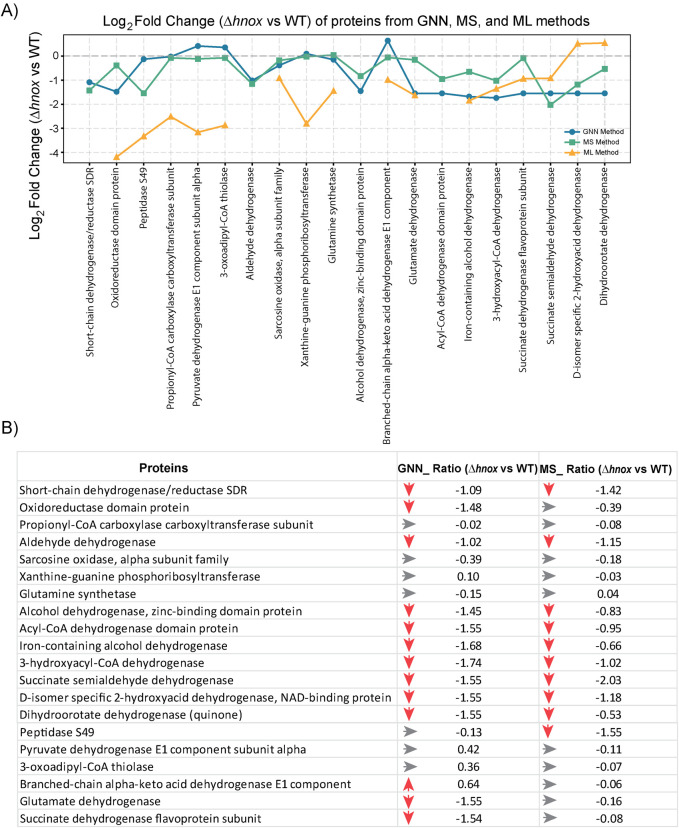
Comparison of predicted and experimental proteome expression (*Δhnox* vs. WT) at OD_600_ = 2.0. **(A)** Line plot showing log_2_ fold change of 20 selected proteins as predicted by the GNN model (blue), ML model (orange), and measured by mass spectrometry (MS) (green). **(B)** Table comparing the protein expression changes (*Δhnox* vs. WT) from GNN predictions and MS based experimental data (p < 0.05). Red arrows indicate downregulation, gray arrows represent minimal or no change. Values are given as log_2_ fold changes.

The GNN methodology successfully produced predictions for 20 proteins in both WT and *Δhnox* strains at OD_600_ = 2.0 (Figure 7; Supplementary Table S5). In this analysis, we focused on a targeted metabolomics dataset centered on central carbon metabolism. Since the GNN models were trained on a defined set of metabolites with known pathway associations, the predictions were naturally limited to proteins closely connected to these metabolites. This resulted in a focused set of proteins, reflecting a deliberate strategy to capture biologically relevant changes rather than broad, proteome-wide predictions. A comparison of the predicted log_2_ fold changes (*Δhnox* vs. WT) with the experimentally obtained values shows superior predictive performance of the GNN model over ML (Figure 7; Supplementary Table S5). While all of the models exhibit certain limitations as discussed below, this methodology presents a valuable framework for predicting proteomic dynamics and investigating metabolic regulation within bacterial systems.

## Discussion

It is now thought that most bacterial species inhabit biofilms, and that this is their primary mode of existence (Flemming and Wuertz, 2019). Thus, biofilm formation is among the most common biological processes on earth, yet its molecular underpinnings remain murky, due in part to the tremendous variety of structures, signaling pathways, and metabolic remodeling accompanying biofilm formation in different species. Biofilm formation in *Paracoccus dentirificans* is unusual for several reasons as discussed above and provides an opportunity to explore the diversity of this process.

Here we have exploited the biofilm-deficient *Δhnox* strain to track biological changes during growth that may promote or repress biofilm formation using a multi-omics approach. The results suggest that early, H-NOX-dependent signaling events tune metabolism to allow biofilm initiation and development. The genomic context of *hnox* suggests that this may occur by inhibition of c-di-GMP synthesis. As cell densities increase, quorum sensing promotes biofilm dispersal in WT *P. denitrificans*. Below, we discuss metabolomics and proteomics changes and how they are correlated at these different growth phases. We also provide an example of how the integration of metabolomics and proteomics data with ML and GNN-based models enables prediction of proteomic shifts.

### H-NOX signaling at low cell density

The role of H-NOX proteins as NO-sensors regulating biofilm formation and dispersal in various species has been extensively reviewed (Plate and Marletta, 2012; Williams et al., 2018; Bacon et al., 2017; Guo and Marletta, 2019; Williams and Boon, 2019; Lee-Lopez and Yukl, 2022). While there are species-specific differences, a common mechanism is that NO-bound H-NOX regulates c-di-GMP levels either by interacting directly with DGC and/or phosphodiesterase (PDE) enzymes or indirectly by regulating kinases that phosphorylate them. The former pathway is used in *Legionella pneumophila* (Carlson et al., 2010) and *Shewanella woodyi* (Liu et al., 2012) where NO-bound H-NOX represses c-di-GMP production or stimulates its degradation, thereby favoring biofilm dispersal. NO-bound H-NOX in *Shewanella oneidensis* and *Vibrio cholerae* inhibits a His kinase, which phosphorylates and activates a PDE (Plate and Marletta, 2012). By inhibiting an activator of PDE, H-NOX signaling increases intracellular c-di-GMP and favors biofilm formation. Either case conforms with the prevailing view that c-di-GMP is a biofilm promoter. The opposite seems to be true in *P. denitrificans* where deletion of *dgc* genes leads to a hyperbiofilm phenotype (Kumar and Spiro, 2017). By this model, H-NOX inhibits c-di-GMP synthesis by the neighboring *dgcA* gene product, thus promoting biofilm formation. Unfortunately, we have been unable to support this mechanism by direct c-di-GMP quantitation. Extraction of this compound from *P. denitrificans* is consistently below the detection limit for either mass spectrometry or ELISA workflows in our hands.

**FIGURE 8:**
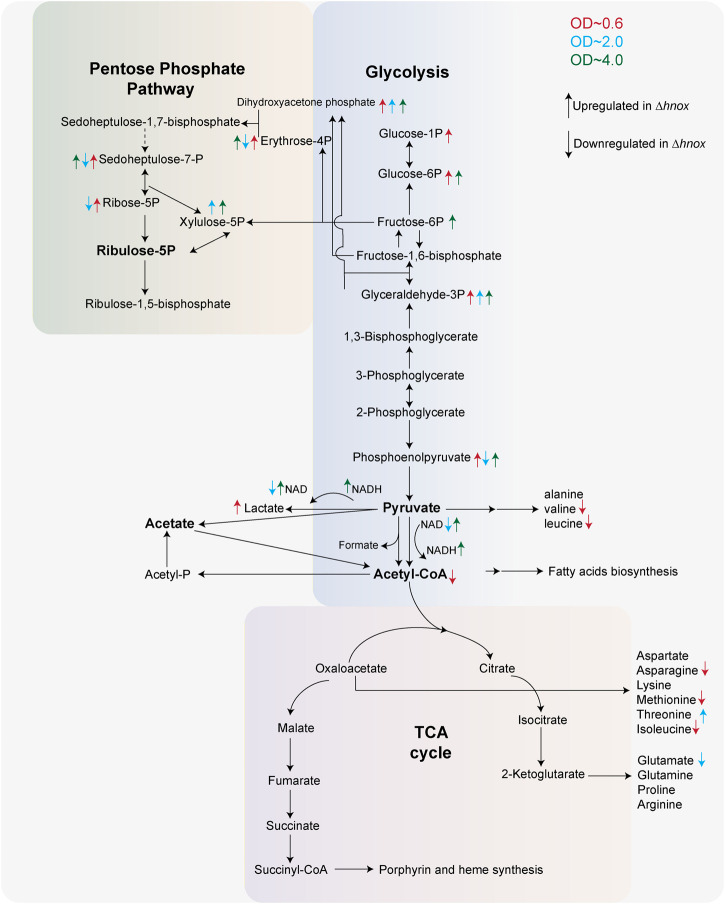
Central metabolic remodeling in Δ*hnox Paracoccus denitrificans.* Central carbon metabolism in *P. denitrificans* highlighting metabolite changes in the *Δhnox* mutant across three growth phases (OD_600_ = 0.6, 2.0, and 4.0). Arrows colored according to OD_600_ indicate significantly increased (↑) or decreased (↓) abundance in *Δhnox* compared to WT.

Whether through c-di-GMP signaling or by other means, we show here that H-NOX signaling influences central carbon metabolism in *P. denitrificans* at OD_600_ = 0.6, prior to high AHL production (Islam et al., 2024). Specifically, we see a notable decrease of abundance in multiple amino acids and acetyl-CoA and a corresponding increase in PPP and glycolysis intermediates in the *Δhnox* strain. Further, we observe detectable pyruvate only in WT at this OD, consistent with our previous observation of decreased pyruvate in *Δhnox*, and an increased abundance of lactate in the mutant. All of this points to inhibition of the last step of glycolysis catalyzed by pyruvate kinase (PK) and/or a transition to fermentative metabolism (Figure 8). Depletion of pyruvate or inactivation of lactate dehydrogenase (LDH) has been shown to impair biofilm formation in *Pseudomonas aeruginosa*, an effect thought to stem from the necessity of redox-balancing in the reductive stress environment found within biofilms (Petrova et al., 2012; Goodwine et al., 2019). Further, since it catalyzes an irreversible step in glycolysis, PK is allosterically regulated, even in bacteria (Sakai et al., 1986; Zoraghi et al., 2010; She et al., 2025).

Our previous proteomics data showed downregulation of both carbohydrate and amino acid transporters in the mutant strain as well as components of the f_0_f_1_ ATP synthase (Islam et al., 2024). Additionally, a downregulation of an oxoacid dehydrogenase analogous to pyruvate dehydrogenase was noted, albeit with an unconfirmed function. These changes correlate with metabolomics changes, although not always in predictable ways. What was not observed was a change to any enzyme convincingly annotated as a LDH or PK. Nevertheless, these enzymes may still be regulated post-translationally through mechanisms that depend on H-NOX and potentially c-di-GMP signaling to facilitate biofilm formation and maintenance.

### H-NOX and quorum sensing at high cell density

Several studies have investigated quorum sensing in *P. denitrificans* and its influence on biofilm formation. At high cell densities, C16 AHL is synthesized through the enzyme PdnI and detected by the transcriptional regulator PdeR (Zhang et al., 2018). The PdeR regulon comprises hundreds of genes including those involved in iron acquisition and ATP synthesis (Wang N. et al., 2022) as well as *pdeI* and *pdeR* themselves. Deletion of *pdeI* or treatment with exogenous C16 HSL inhibits biofilm formation (Toyofuku et al., 2017; Morinaga et al., 2018) while overexpression of PdeR enhances it (Wang N. et al., 2022). At OD_600_ = 2 and 4, C16 HSL levels rise precipitously (Islam et al., 2024), correlating with decreased relative biofilm in WT *P. denitrificans* (Figure 1B) and supporting a role in biofilm dispersal for QS in this species. These levels are higher in the *Δhnox* strain, which exhibits roughly the same low relative level of biofilm across all cell densities. QS and c-di-GMP signaling are tightly integrated in other species (Römling et al., 2013), suggesting that dysregulation of c-di-GMP production in the mutant strain may drive subsequent dysregulation of QS.

Given the large size of the PdeR regulon, metabolomic and proteomic changes at high cell densities would presumably be dominated by QS targets. However, we did not observe any of the iron transporters shown to be differentially transcribed in the *pdeR* or *pdeI* deletion strains (Zhang et al., 2018) in our proteomics dataset at OD_600_ = 2.0. Although PdeR and BapA were identified, neither was differentially abundant between WT and *Δhnox*. As the name implies, QS occurs above a threshold concentration (Platt and Fuqua, 2010; Waters and Bassler, 2005), and the lack changes in expression to known PdeR regulon proteins may indicate that this threshold has been reached in both strains. However, changes in metabolic enzymes and transporters are still observed. Perhaps most conspicuous among these are a decrease in a number of metabolic dehydrogenase enzymes including acyl-CoA and 3-hydroxyacyl-CoA dehydrogenases involved in fatty acid beta-oxidation. These are intriguing in that they suggest changes to fatty acid metabolism not captured in our targeted metabolomics approach.

In the metabolomics data, we observe increased abundance of glyceraldehyde 3-phosphate, dihydroxyacetone phosphate, and xylulose 5-phosphate in *Δhnox* strains at OD_600_ = 2.0. These are intermediates of glycolysis and/or PPP (Figure 8), continuing the trend observed at OD_600_ = 0.6, although other intermediates in these pathways are modestly decreased in abundance at OD_600_ = 2.0. In *Burkholderia glumae*, transcription of glycolytic enzymes as well as abundance of glycolytic and PPP metabolites are decreased by quorum sensing (An et al., 2014). However, it is worth noting that AHL QS in the closely related *Burkholderia cenocepacia* stimulates biofilm (Aguilar et al., 2014) in contrast to what we observe in *P. denitrificans*. We continue to observe higher abundances of glycolytic and PPP metabolites at OD_600_ = 4.0 with concomitant increases in redox cofactors NAD(H), FMN, and NADP. These may indicate a disruption in redox homeostasis that could also impair biofilm formation and maintenance.

### AI-driven proteomics prediction and its biological implications

This work illustrates that bacterial cell density has a substantial influence on central metabolism, and relatively small changes in metabolism can have significant consequences for phenotypes such as biofilm formation. This is especially evident from how the metabolic profiles of WT and *Δhnox P. denitrificans* cluster tightly at various OD values (Figure 5A), yet these strains exhibit dramatic differences in biofilm formation. Controlling for cell density is clearly important when drawing mechanistic conclusions from -omics data, making predictive tools especially valuable in this context. The datasets collected here and in our previous work (Islam et al., 2024) provide an excellent opportunity to test the ability of ML and GNN models to predict protein expression from existing -omics data from a different cell density. The GNN model in particular demonstrated significant predictive ability for a small subset of proteins identified as differentially abundant by mass spectrometry. Interestingly, the majority of predicted proteins are NAD(H)-dependent dehydrogenases, suggesting that the observed differential abundance of this cofactor at OD_600_ = 2.0 drives predictive decisions in the model. Xanthine-guanine phosphoribosyl transferase is also identified, which transfers a phosphoribosyl group to guanine or xanthine, resulting in the formation of GMP or XMP, respectively. Presumably, changes in guanine and xanthine nucleotides XMP, GTP, and cGMP drives identification of this enzyme, which was correctly predicted to be unchanged in abundance.

The examination of various ML and AI models yields significant insights applicable to multi-omics research. Models characterized by simplicity, such as Ridge regression, demonstrate robust performance in small-sample contexts by reducing overfitting. In contrast, more intricate methodologies, such as GNNs have the capacity to leverage pathway topology and identify non-linear relationships, thereby attaining enhanced biological concordance. This underscores the necessity of employing benchmarking across various models, instead of depending on a singular methodology, as a recommended practice for predictive omics research. Comparative analyses assure the transparency of methodological choices and ensure that conclusions derived from AI-driven models are reliable and biologically meaningful. For this data, the GNN-predicted fold changes agreed best with MS-validated data, exhibiting a moderate positive correlation (Pearson’s r = 0.47). While this model captures part of the biological signal, predictive power remains limited by sample size and model simplicity. The small sample size also heightens the risk of overfitting, especially in deep learning approaches like graph neural networks. We employed leave-one-out cross-validation to optimize data utilization, while recognizing its established variance in small-sample contexts. Consequently, our ML analysis should be viewed as a proof-of-concept demonstration of feasibility rather than as a conclusive predictive model. The observed concordance between predicted and experimentally measured fold-changes indicates the potential of this integrative approach, while emphasizing the necessity for larger datasets and additional time points in future research.

## Conclusion

This comprehensive analysis of metabolomics and proteomics, augmented by AI-based modeling, expands on the prior literature of unusual biofilm formation and signaling in *P. denitrificans*. H-NOX influences central carbon metabolism at relatively low cell densities, potentially by regulating c-di-GMP metabolism. Deletion of *hnox* results in decreased amino acid abundance and increases in glycolytic and PPP metabolites, potentially indicating inhibition of late glycolytic enzymes and/or promotion of fermentation. QS occurs at higher cell densities, which we have confirmed to promote biofilm dispersal in this organism. Despite significant differences in the quantity of C16 AHL between the strains at these densities, we did not observe differential expression of known QS targets, suggesting that signaling threshold had been reached in both strains. However, changes to metabolic enzymes and metabolites persisted, particularly in PPP and glycolytic pathways. Finally, metabolomic and proteomic data sets were used to test AI/ML models for predicting proteome changes from metabolomic data. Despite limitations of sample size, a GNN model demonstrated moderate correlation between predicted and experimental fold changes, providing a proof-of-concept validation for using metabolomic data to anticipate proteomic outcomes. Collectively, these findings enhance our understanding of metabolic regulation in *Paracoccus denitrificans* that may be useful for future applications in microbial engineering and environmental biotechnology.

## Data Availability

The proteomics data presented in the study have been deposited to the ProteomeXchange Consortium via the jPOSTrepo partner repository, accession numbers PXD066973 and JPST003986. The code for ML and GNN models can be accessed from https://github.com/SIslam007/Metabolomics-and-Proteomics-projects-with-ML-and-DL.git.
