# Supplementary material for: Unraveling metabolic reprogramming in Δhnox Paracoccus denitrificans: a time-resolved metabolomics and AI-Powered proteome modeling approach
Source: Front Mol Biosci. 2025 Oct 21;12:1679650. doi: 10.3389/fmolb.2025.1679650 (PMC12582929; doi:10.3389/fmolb.2025.1679650)
Supplement: Supplementary file 6 [file DataSheet1.docx]

**Supplemental Information**

**Unraveling Metabolic Reprogramming in Δhnox Paracoccus denitrificans: A Time-Resolved Metabolomics and AI-Powered Proteome Modeling Approach**

Md Shariful Islam^1^, Aishat Alatishe^1^, William Bahureksa^2^, Erik Yukl^1^***

*^1^* Department of Chemistry and Biochemistry, New Mexico State University, Las Cruces, New Mexico, USA.

*^2^* Chemical Analysis & Instrumentation Laboratory Research Cores Program, New Mexico State University, Las Cruces, New Mexico, USA.

^*^Correspondence: Tel. 575 646 3176; Fax. 575 646 2649; E-mail: [etyukl@nmsu.edu](mailto:wilmo004@umn.edu)

**
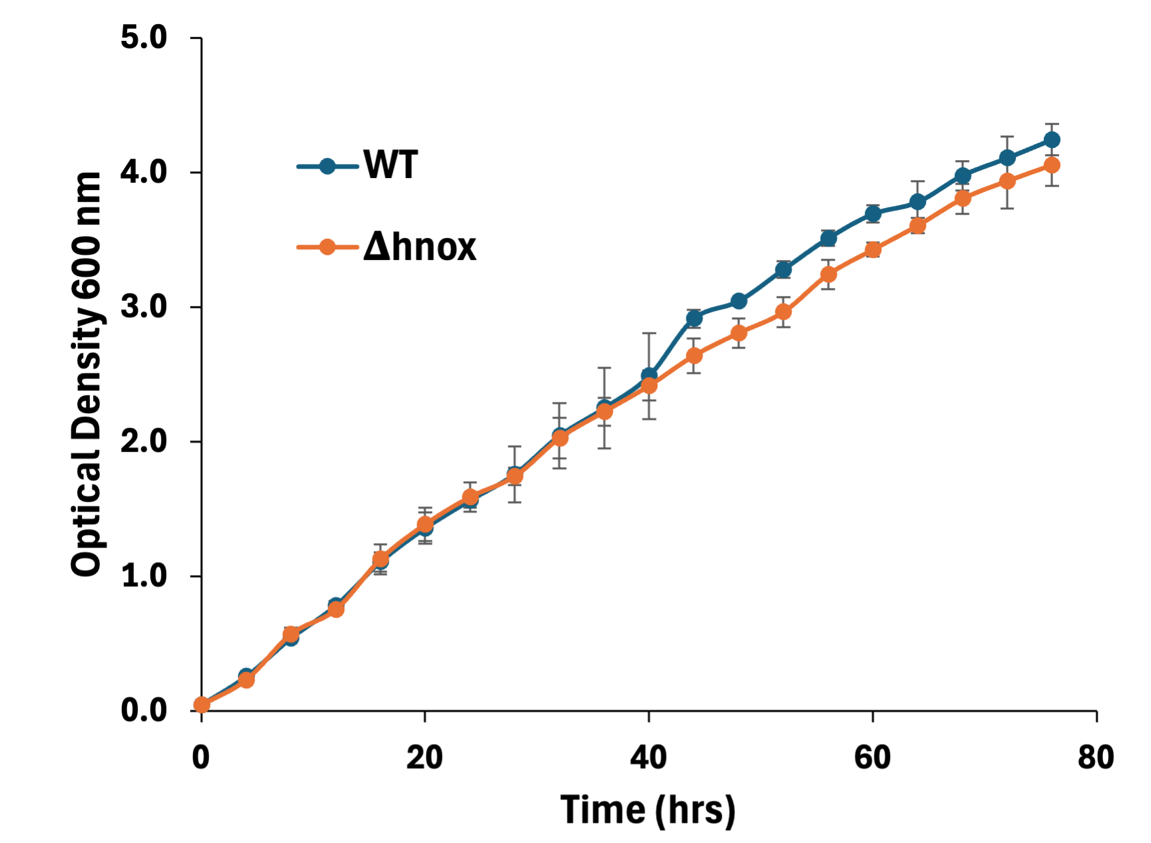
**

**Figure S1:** Growth of WT and *Δhnox P. denitrificans* in LB media. Error bars represent standard deviation of 3 biological replicates.


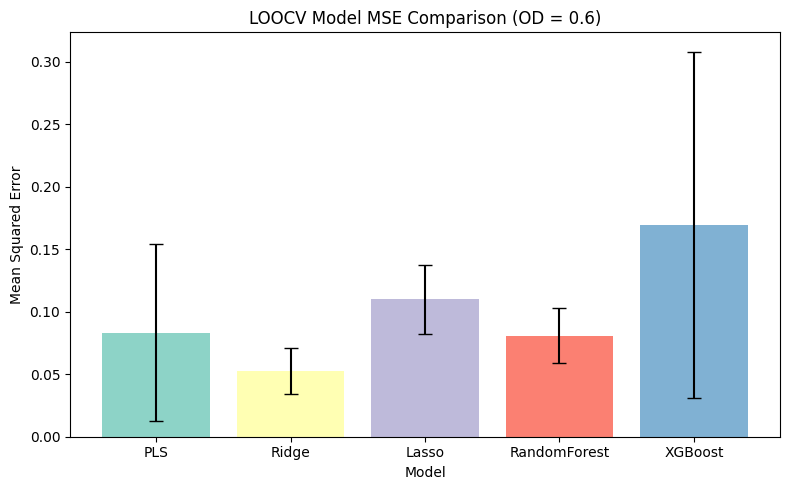


**Figure S2:** *Comparison of machine learning model performance using Leave-One-Out Cross-Validation (LOOCV) at OD = 0.6.* Mean Squared Error (MSE) values are shown for Partial Least Squares (PLS), Ridge regression, Lasso regression, Random Forest, and XGBoost models. Bars represent mean MSE, and error bars indicate standard deviation across cross-validation folds. Lower MSE values correspond to better predictive performance.
